# Service quality: perspective of people with type 2 diabetes mellitus and hypertension in rural and urban public primary healthcare centers in Iran

**DOI:** 10.1186/s12913-024-10854-y

**Published:** 2024-04-24

**Authors:** Shabnam Iezadi, Kamal Gholipour, Jabraeil Sherbafi, Sama Behpaie, Nazli soltani, Mohsen Pasha, Javad Farahishahgoli

**Affiliations:** 1 Tabriz Health Services Management Research Center, Department of Health Policy and Management, School of Management and Medical Informatics, Tabriz University of Medical Sciences, Tabriz, Iran; 2 East Azerbaijan Provincial Health Centre, Tabriz University of Medical Sciences, Tabriz, Iran; 3 Student Research Committee, Department of Health Policy and Management, School of Management and Medical Informatics, Tabriz University of Medical Sciences, Tabriz, Iran

**Keywords:** Diabetes Mellitus, type 2, Hypertension, Quality of Health Care, Service Quality, patient satisfaction

## Abstract

**Objective:**

This study aimed to assess the service quality (SQ) for Type 2 diabetes mellitus (T2DM) and hypertension in primary healthcare settings from the perspective of service users in Iran.

**Methods:**

The Cross-sectional study was conducted from January to March 2020 in urban and rural public health centers in the East Azerbaijan province of Iran. A total of 561 individuals aged 18 or above with either or both conditions of T2DM and hypertension were eligible to participate in the study. The study employed a two-step stratified sampling method in East Azerbaijan province, Iran. A validated questionnaire assessed SQ. Data were analyzed using One-way ANOVA and multiple linear regression statistical models in STATA-17.

**Results:**

Among the 561 individuals who participated in the study 176 (31.3%) were individuals with hypertension, 165 (29.4%) with T2DM, and 220 (39.2%) with both hypertension and T2DM mutually. The participants’ anthropometric indicators and biochemical characteristics showed that the mean Fasting Blood Glucose (FBG) in individuals with T2DM was 174.4 (Standard deviation (SD) = 73.57) in patients with T2DM without hypertension and 159.4 (SD = 65.46) in patients with both T2DM and hypertension. The total SQ scores were 82.37 (SD = 12.19), 82.48 (SD = 12.45), and 81.69 (SD = 11.75) for hypertension, T2DM, and both conditions, respectively. Among people with hypertension and without diabetes, those who had specific service providers had higher SQ scores (b = 7.03; *p* = 0.001) compared to their peers who did not have specific service providers. Those who resided in rural areas had lower SQ scores (b = -6.07; *p* = 0.020) compared to their counterparts in urban areas. In the group of patients with T2DM and without hypertension, those who were living in non-metropolitan cities reported greater SQ scores compared to patients in metropolitan areas (b = 5.09; *p* = 0.038). Additionally, a one-point increase in self-management total score was related with a 0.13-point decrease in SQ score (*P* = 0.018). In the group of people with both hypertension and T2DM, those who had specific service providers had higher SQ scores (b = 8.32; *p* < 0.001) compared to the group without specific service providers.

**Conclusion:**

Study reveals gaps in T2DM and hypertension care quality despite routine check-ups. Higher SQ correlates with better self-care. Improving service quality in primary healthcare settings necessitates a comprehensive approach that prioritizes patient empowerment, continuity of care, and equitable access to services, particularly for vulnerable populations in rural areas.

**Supplementary Information:**

The online version contains supplementary material available at 10.1186/s12913-024-10854-y.

**Table:** 

**Article Summary**
*Strengths and limitations of this study*:
• Comprehensive representation with a commendable inclusion of both urban and rural public health centers, providing a holistic view of service quality.
• Credibility enhanced by the use of a valid and reliable questionnaire to assess service quality.
• Challenges linked to infrastructure weakness and health information system limitations impacted data extraction, potentially affecting result accuracy.
• Inclusion of urban and rural centers, while diverse, raises concerns about generalizability due to study design and sampling method. Caution is needed in the broad application of findings.

## Background

Diabetes and hypertension, recognized as major contributors to premature mortality, stand as primary risk factors for heart attacks, strokes, and kidney diseases [1, 2]. Diabetes, in particular, may result in blindness and lower limb amputations [1]. The prevalence of diabetes is on the rise globally, especially in low- and middle-income countries (LMICs), where approximately two-thirds of individuals with hypertension reside [3, 4]. Existing literature underscores the high prevalence of Type 2 Diabetes Mellitus (T2DM) and/or hypertension in Iran, akin to other LMICs, posing substantial threats to patients and healthcare systems if not effectively managed [4–6]. Alarmingly, evidence indicates that the rates of treatment and control for both T2DM and hypertension in Iran are notably lower than in higher-income countries, magnifying the potential for severe consequences [4, 7].

The global healthcare community has increasingly emphasized the importance of quality of care since the Institute of Medicine’s landmark publication, “Crossing the Quality Chasm,” urging essential changes to bridge the quality gap by the end of the 21st century [8]. Despite these efforts, many health systems, particularly those in LMICs, grapple with low-quality care [8]. Poor quality of care stands out as a significant factor contributing to inadequate control of hypertension and T2DM [9, 10]. Studies have consistently shown a positive correlation between receiving high-quality care for diabetic or hypertensive conditions and achieving better health outcomes [9–11]. Consequently, gaining a deeper understanding of the quality of care provided to patients with T2DM and/or hypertension is crucial for effective community management.

Assessing quality is a foundational step toward enhancing care for individuals with chronic health conditions [12]. Quality of care can be assessed from various perspectives, including technical and service quality. Technical quality assesses the adherence of services to established guidelines [13], while service quality examines the overall quality of services provided to patients [14]. SQ primarily describes how the received care is perceived and influenced by various factors such as physical, social, and cultural contexts, as well as aspects like accessibility, respect, and confidentiality [14]. Most studies examining the quality of T2DM and/or hypertension care have predominantly focused on technical aspects, with only a handful exploring service quality [15, 16]. Notably, despite the higher prevalence of T2DM and hypertension in LMICs, the majority of studies examining service quality for these conditions originate from high-income countries, underscoring the imperative for additional research in LMICs [3, 4, 17]. This study aims to fill this gap by assessing service quality for T2DM and hypertension in primary healthcare settings from the perspective of service users in Iran.

## Methods

### Study design

This cross-sectional study was conducted from January to March 2020 in the East Azerbaijan province of Iran. We adhered to The Strengthening the Reporting of Observational Studies in Epidemiology (STROBE) guidelines to prepare our study report [18].

### Study settings and participants

The target population included individuals seeking healthcare from health centers in the East-Azerbaijan province of Iran. Eligible participants were aged 18 or above, diagnosed with T2DM and/or hypertension at least 12 months before data collection.

We employed a two-step stratified sampling method. Initially, all 20 districts in East-Azerbaijan province were categorized into metropolitan, densely populated urban, and predominantly rural areas. Subsequently, we randomly selected districts (Tabriz, Marand, Bostanabad, Varzaqhan, Ajabshir) and health centers within those districts. Participants were then randomly selected from lists of eligible individuals in each selected health center.

### Sample size calculation

Using the G-Power program (Heinrich-Heine-Universität Düsseldorf, Düsseldorf, Germany), we calculated a sample size of 637 based on 95% power, 0.05 α and an effect size of 0.07 to consider the stratified sampling, considering a linear regression test based on a fixed model.

### Participants’ recruitment

Health workers in selected centers communicated with potential participants during routine care visits. They explained the study’s purpose, introduced the research team, and obtained written consent from willing participants. To safeguard privacy, participants could complete the anonymous questionnaire in a separate room.

### Data collection

Data was collected from January to March 2020 using a standard SQ questionnaire (the validity and reliability were already approved in similar contexts) [19–21]. The questionnaire included four main parts. The first part consisted of the demographic characteristics (age, gender, place of birth, current residency, language, employment status, health insurance status, and education level). The second part encompassed questions related to disease conditions (medical history, type of treatment, complications, and smoking status), and the third part contained questions related to self-management conditions. The final part included 37 questions in 13 dimensions of service quality (SQ), including choice of care provider (2 questions), communication (4 questions), autonomy (4 questions ), availability of support groups (3 questions), continuum of care (2 questions), basic amenities (4 questions), dignity (4 questions), timeliness (4 questions), safety (2 questions), prevention services (2 questions), accessibility of services (2 questions), confidentiality (2 questions) and dietary counseling (2 questions).

Despite previous validation, the face validity of the questionnaire was reviewed and confirmed by health management specialists and cardiologists at Tabriz University of Medical Sciences, and its reliability was confirmed according to the Cronbach’s alpha coefficient (α = 0.81) in a pilot study on 30 participants. We recruited 13 participants from urban and 17 participants from rural center in pilot study. Cronbach’s alpha coefficient ranged from (α = 0.67) for “timeliness”, to (α = 0.83) for “dietary counseling”. Additionally, according to previous studies, an SQ score of less than “nine” indicates a failure in the quality of care and a significant gap for improvement [19–21]. We excluded the participants of the pilot study from the main sample size to avoid any bias.

### Data analysis

For each question, participants were asked to report the importance of the item and their perception of the quality of care they had received about that item (performance) during the last 12 months. Questions related to the importance of the SQ items were scored on a four-point Likert scale, which was then scaled from 1 to 10 (1 = not important, 3 = somehow important, 6 = important, and 10 = very important). Questions related to the perceived performance of services were also scored on a four-point scale ranging from ‘‘never, sometimes, usually, and always’’ or ‘‘poor, fair, good, and excellent’’. For analysis, this scale was dichotomized, say, 0 = usually/always or good/excellent and 1 = never/sometimes or poor/fair [22, 23].

An overall measure of SQ was calculated for each SQ dimension by combining the importance and performance scores using the following formula [22, 23]:

Service Quality = 10 – (importance × performance).

SQ score ranges from 0 (worse) to 10 (best). The SQ score of each dimension was calculated as mean SQ scores of that dimension’s questions and total SQ was calculated as mean SQ scores of all 37 questions. Finally, the service quality score was reported on a scale of 0-100.We assessed and confirmed the normality of data by one sample Kolmogorov–Smirnov test (*n* = 561, Z = 0.07, *P*_Value = 0.06). We reported frequencies and percentages for categorical variables and mean and standard deviation for the numerical variables, including, age and SQ score and its dimensions. We used the One-Way ANOVA test to analyze the differences between the anthropometric indices and biochemical characteristics and dimensions of SQ in categorical variables.

We employed a two-step linear regression analysis as the entering method for our data analysis. Variables identified as related with Service Quality (SQ) in the univariate analysis were included in the multiple linear regression model. The significance thresholds for the entry and removal of variables in the stepwise regression model were set at 0.05 and 0.25, respectively. Additionally, age, education, continuous care by specialists, and self-evaluation of disease were included as control variables.

To ensure the validity of our regression analysis, we conducted several checks. Normality of residuals was assessed and confirmed through the normal probability plot, while the homogeneity of residual variances was verified via the residual versus predicted values plot. We further ensured residual independence and addressed multicollinearity by employing Durbin-Watson Statistics and Variance Inflation Factor, respectively. These steps were taken to fulfill all assumptions of multiple linear regression. Also, reference categories in regression analysis were selected based on the research team’s theoretical interest and previous studies.

Statistical significance was determined at a *p*-value threshold of < 0.05. The data were meticulously analyzed using the STATA version 17 (StatsCorp, College Station, TX, USA).

## Results

Among the 637 contacted patients, an impressive 561 individuals participated in the study, reflecting a robust response rate of 91.1%. The majority of participants were female (69%), hailing from metropolitan areas (36%), predominantly speaking Azeri (94%), unemployed (74%), lacking supplementary health insurance (65%), and reporting illiteracy (41%) (Table 1).

**Table 1:** Self-reported socio-demographics of study participants with Type2 Diabetes Mellitus and Hypertension

Characteristics		Hypertension		T2DM		Hypertension & T2DM		*P*-Value
		N	%	N	%	N	%	
**Gender**	Male	48	27.3	60	36.4	62	28.2	0.129
	Female	128	72.7	105	63.6	158	71.8	
**Age**	25–40 years	22	12.8	24	14.6	4	1.8	< 0.001
	40–60 years	91	52.9	94	57.3	97	44.7	
	60–88 years	59	34.3	46	28.0	116	53.4	
**Residence Areas**	Metropolitan area	62	36.9	58	36.0	85	38.8	0.997
	Urban areas	53	31.5	54	33.5	69	31.5	
	Village	53	31.5	49	30.4	65	29.7	
**Language**	Azeri	167	98.8	150	97.4	211	99.1	0.323
	Farsi	2	1.2	4	2.6	2	1	
**Employment status**	Employed	49	28.0	48	29.3	48	21.8	0.318
	Unemployed	126	72.0	116	70.7	171	77.7	
**Having health insurance**	Yes	169	95.5	161	97.6	216	98.2	0.254
	No	8	4.5	4	2.4	4	1.8	
**Having Supplementary health insurance**	Yes	52	31.9	43	29.3	57	27.4	0.640
	No	111	68.1	104	70.7	151	72.6	
**Education level**	Illiterate	63	35.6	62	37.6	108	49.1	0.019
	Under diploma	71	40.1	76	46.0	81	36.9	
	Diploma	27	15.3	15	9.1	21	9.5	
	Tertiary	16	9.0	11	6.6	9	4.1	

he anthropometric indices and biochemical characteristics of the participants revealed a predominant occurrence of overweight status. Notably, the mean Fasting Blood Glucose (FBG) levels in individuals with Type 2 Diabetes Mellitus (T2DM) were elevated, measuring 174.4 (73.57) in patients with T2DM without hypertension and 159.4 (65.46) in patients with both T2DM and Hypertension. Additional details regarding the participants’ anthropometric indices and biochemical characteristics can be found in Table 2.

**Table 2:** Analysis of differences in the participants’ anthropometric indices and biochemical characteristics between people with T2DM and hypertension and both conditions

Anthropometric indices and biochemical characteristics*	Hypertension		T2DM		Hypertension & T2DM		*P*-Value**
	Mean	SD	Mean	SD	Mean	SD	
BMI	29.36	5.153	29.88	4.5	29.88	4.5	0.280
Weight (KG)	75.29	12.95	74.43	14.2	76.39	12.64	0.392
FBG***	-	-	174.4	73.57	159.4	65.46	0.086
HbA1c***	-	-	8.00	1.84	8.66	8.13	0.561
Systolic BP (mm Hg)	12.55	1.20	12.04	1.18	12.5	1.26	0.012
Diastolic BP (mm Hg)	7.85	0.82	8.32	7.20	7.87	0.89	0.650

*Based on electronic health record, ** One-Way ANOVA, *** Independent Samples Test

Abbreviations: T2DM: Type2 Diabetes Mellitus; BMI: Body Mass Index; FBG: Fasting Blood Glucose; HbA1c: Hemoglobin A1C; BP: Blood Pressure

The findings regarding self-reported hypertension self-management status indicated that among individuals with hypertension without Type 2 Diabetes Mellitus (T2DM), the majority adhered to the “regular use of prescription drugs” (approximately 94%). Conversely, “regular blood pressure measurement at home” was the least adhered-to item, with an adherence rate of around 61%. In contrast, among patients with both T2DM and hypertension, a substantial proportion reported adherence to a “recommended diet” (approximately 90%) and being “aware of the side effects of high blood pressure” (roughly 88%). The results of Fisher’s Exact Test and Independent Samples Test demonstrated no statistically significant relationship between hypertension self-management status and the presence of T2DM among individuals with hypertension, neither for sub-items nor for the total score. Comprehensive details on the hypertension self-management status of participants are presented in Table 3.

**Table 3:** Analysis of differences in Hypertension self-management status based on the participants’ self-reported data between people with Hypertension and Simultaneous Hypertension & T2DM

Self-management status	Hypertension		Hypertension & T2DM		*P*-Value*
	N	%	N	%	
Family history of high blood pressure	114	65.9	146	67	0.830
See the caregiver on time and regularly	131	75.3	171	78.8	0.467
Regular blood pressure measurement at home	106	60.9	120	55	0.259
See your doctor regularly despite your blood pressure being normal	130	74.7	160	73.4	0.817
Regular use of prescription drugs	163	93.7	207	96.3	0.248
Adhere to the recommended diet	151	86.8	191	88	0.760
Knowing the side effects of high blood pressure	151	86.8	194	89.8	0.426
	**Mean**	**SD**	**Mean**	**SD**	***P***-Value**
BP self-management total score (0-100)	79.7	20.3	80.1	19.6	0.838

* Fisher’s Exact Test, ** Independent Samples Test

Abbreviations: T2DM: Type2 Diabetes Mellitus; SD: Standard Deviation

The self-reported Type 2 Diabetes Mellitus (T2DM) self-management status revealed that the majority of participants adhered to the “regular use of prescription drugs” (approximately 97%). Conversely, “regular glucose measurement at home” emerged as the least adhered-to items, with adherence rates of approximately 58% among patients with T2DM without hypertension and 47% among patients with both T2DM and hypertension. A comprehensive overview of the T2DM self-management status of patients is presented in Table 4.

**Table 4:** Analysis of differences in T2DM self-management status based on the participants’ self-reported data between people with Hypertension and Simultaneous Hypertension & T2DM

Self-management condition	T2DM		Hypertension & T2DM		*P*-Value*
	N	%	N	%	
Family history of T2DM	87	53.0	127	58	0.351
See the caregiver on time and regularly	128	78	177	80.8	0.523
Regular blood glucose measurement at home	96	58.5	103	47	0.030
See your doctor regularly despite your blood glucose being normal	109	66.5	157	71.7	0.313
Regular use of prescription drugs	158	97.5	210	96.3	0.568
Adhere to the recommended diet	139	84.8	188	85.8	0.772
Knowing the side effects of T2DM	144	87.8	193	88.5	0.873
	**Mean**	**SD**	**Mean**	**SD**	***P***-Value**
T2DM self-management total score (0-100)	78.8	21.1	78.3	20.0	0.830

* Fisher’s Exact Test, ** Independent Samples Test

Abbreviations: T2DM: Type2 Diabetes Mellitus; SD: Standard Deviation

Among all 13 dimensions of Service Quality (SQ), confidentiality and dignity exhibited the highest scores across all groups. The total SQ scores were 82.37 (12.19), 82.48 (12.45), and 81.69 (11.75) for hypertension, Type 2 Diabetes Mellitus (T2DM), and both conditions (Hypertension & T2DM), respectively. Notably, there were no statistically significant differences in total SQ scores between the groups (*P* = 0.780). Detailed results of SQ scores for each group are presented in Table 5.

**Table 5:** Analysis of differences in SQ Score and its dimensions between people with T2DM, Hypertension, and Simultaneous Hypertension & T2DM

Service Quality Dimensions	Hypertension SQ score*		T2DM SQ score*		Hypertension & T2DM SQ score*		*P*-Value**
	Mean (SD)		Mean (SD)		Mean (SD)		
Choice of care provider	75.71	32.3	72.99	34.45	76.56	31.34	0.559
Communication	88.11	20.59	85.98	22.24	87.17	19.68	0.637
Autonomy	86.97	19.22	89.86	16.17	89.17	17.03	0.273
Availability of support group	60.72	33.04	60.77	32.21	58.33	32.07	0.690
Continuity of care	84.46	24.57	89.42	22.13	84.91	24.97	0.109
Basic amenities	91.69	16.87	89.62	18.26	88.8	19.17	0.284
Dignity	92.79	16.91	92.1	15.1	93.76	13.87	0.563
Timeliness	79.51	23.91	76.14	25.4	78.99	21.77	0.364
Safety	84.66	29.01	88.29	24.34	82.47	29.27	0.128
Prevention	70.8	38.98	76.71	34.82	70.46	36.45	0.204
Accessibility	78.91	26.61	77.07	26.86	72.64	29.39	0.070
Confidentiality	94.54	15.19	94.82	14.95	92.9	18.81	0.465
Diet	63	37.73	64.3	38.09	65.5	39.23	0.828
Total SQ score	82.37	12.19	82.48	12.45	81.69	11.75	0.780

‡ Importance score: Range between 0 (not important) and 10 (very important)

† Performance score: Range between 0 (good) and 1 (poor)

* Service Quality score: 100 is the best and 0 is the worst score

** One-Way ANOVA

Abbreviations: SQ: Service Quality; T2DM: Type2 Diabetes Mellitus; SD: Standard Deviation

The Multiple Regression model results unveiled several relationships with Service Quality (SQ) scores in different patient groups. Among individuals with hypertension and without diabetes, those with specific service providers demonstrated higher SQ scores (b = 7.03; *p* < 0.001) compared to those without specific service providers. Moreover, individuals in rural areas with hypertension and without diabetes exhibited lower SQ scores (b = -6.07; *p* < 0.05) than their urban counterparts.

In the group of patients with Type 2 Diabetes Mellitus (T2DM) and without hypertension, those residing in non-metropolitan cities reported higher SQ scores compared to patients in metropolitan areas (b = 5.09; *p* < 0.05). Additionally, a one-point increase in self-management total score was related with a 0.13-point decrease in SQ score (*P* < 0.05).

For people with both hypertension and T2DM, those with specific service providers demonstrated higher SQ scores (b = 8.32; *p* < 0.001) compared to those without specific service providers. Patients with both conditions who had a diabetes history of over 10 years exhibited higher SQ scores than those with less than two years of diabetes history (b = 4.47; *p* < 0.05).

**Table 6:** Results of Multiple Linear Regression Analysis for Variables Related to total Service Quality score between people with T2DM, Hypertension, and Simultaneous Hypertension & T2DM

Characteristics		Hypertension			T2DM			Hypertension & T2DM		
		b	95% CI		b	95% CI		b	95% CI	
			LB	UB		LB	UB		LB	UB
**Age**		0.03	-0.16	0.23	0.11	-0.08	0.29	-0.09	-0.26	0.07
**Sex**	Male†	1			1			1		
	Female	-0.52	-5.19	4.14	-1.28	-5.94	3.39	-0.93	-5.06	3.20
**Residency**	Metropolitan	1			1			1		
	City	0.99	-3.85	5.84	5.09*	0.28	9.90	1.32	-2.76	5.40
	Village	-6.07*	-11.19	-0.95	-3.18	-8.79	2.44	-1.57	-5.49	2.35
**Education**	Illiterate†	1			1			1		
	Under diploma	-1.95	-6.72	2.82	0.61	-4.35	5.57	0.54	-3.19	4.26
	Diploma	0.07	-7.09	7.22	4.99	-3.87	13.86	-1.61	-8.96	5.75
	Tertiary	-2.65	-10.84	5.54	-2.04	-10.79	6.71	6.30	-2.15	14.75
**specific provider#**	No†	1			1			1		
	Yes	7.03**	2.97	11.09	3.91	-0.27	8.09	8.32**	5.15	11.50
**Smoke/hookah consumption**	No†	1			1			1		
	Yes	9.91	-0.70	20.53	-2.02	-9.05	5.01	-1.17	-8.54	6.21
**BP self-management total score**		0.05	-0.04	0.15				0.02	-0.08	0.12
**T2DM self-management total score**					0.13*	0.02	0.23	0.02	-0.07	0.12
**Duration of T2DM awareness**	Under 2 years†				1			1		
	2–5 years				4.16	-1.27	9.60	-1.99	-6.27	2.27
	5–10 years				-3.95	-9.20	1.30	2.80	-1.35	6.95
	Over 10 years				-0.92	-7.63	5.79	4.47*	0.04	8.90

* *P*value < 0.05; ** *P*value < 0.001

† Reference category

# seeing the same care provider for care during last year

Abbreviations: T2DM: Type2 Diabetes Mellitus; SD: Standard Deviation; LB: Lower Bound; UB: Upper Bound

# seeing the same care provider for care during last year

Abbreviations: T2DM: Type2 Diabetes Mellitus; SD: Standard Deviation; LB: Lower Bound; UB: Upper Bound

## Discussion

### Statement of principal findings

In this study, the evaluation of service quality (SQ) for Type 2 Diabetes Mellitus (T2DM) and hypertension in primary healthcare settings in Iran revealed that SQ scores for participants with T2DM without Hypertension, those with hypertension without T2DM, and those with both conditions were at an average level. The primary weaknesses identified in SQ were related to the availability of support groups, self-care training, and dietary counseling.

In our study, participants reported higher scores for “dignity” and “confidentiality” items in service provision compared to the other dimensions of the SQ, while the lowest score was reported for the availability of support groups. The significant role of the support groups in controlling patients with T2DM and/or hypertension, especially in LMICs with a rising burden of diabetes, is well documented. For example, studies have reported that support groups can enhance diabetes knowledge and psychosocial functioning [24, 25], improve diabetes outcomes [26, 27], and enhance self-management behaviors [27, 28]. Therefore, it is of fundamental importance to take advantage of support groups when providing services for patients with diabetes or other chronic health conditions. However, this principle component of care seems to be ignored in the care process of patients with T2DM and/or hypertension in Iran.

In addition to access to support groups, the dimensions of “nutrition counseling”, “disease prevention services”, and “the right to choose service providers” had the lowest scores among all dimensions of SQ in all three groups of patients. However, a strong body of evidence has shown that due to the important role of nutrition interventions in improving glucose metabolism, weight, BMI, and waist circumference in T2DM [29], nutrition counseling is essential for patients with T2DM [30]. Other studies, on the other hand, have highlighted the role of social interactions in the effective control of T2DM and/or hypertension and in guiding the self-management tasks. For instance, one study showed that risks of uncontrolled hypertension are lower among those with higher social interactions who discuss their health issues with others in a social group [31]. Due to the importance of these elements in care process of the patients with T2DM and/or hypertension, it is critical for the health system to employ plans to monitor the performance of the healthcare provider with regard to service quality of chronic health conditions.

To achieve desirable outcomes in treating patients with T2DM and/or hypertension, healthcare providers need to be very concrete about providing self-management and dietary counseling. Moreover, considering the progressive nature of T2DM and hypertension and the need for constant monitoring of progress and any complications of the disease, it is necessary to provide them with accurate training and self-management advice by the service providers. In addition, the authorities of the health system should take measures to continuously evaluate the status of these services and care in the healthcare center.

Based on the results of this study, the patients’ self-care status was not favorable. Poor performance in implementing self-care programs indicates that healthcare providers may have failed to achieve care goals for patients with chronic conditions. The results of the study also showed that generally the better the self-care status the higher the SQ score. This finding may imply that empowered patients can receive better care from service providers [32].

The results of our study identified that people who received their services from a specific provider reported significantly higher scores for SQ than those without a specific service provider. This highlights the need for stability in service providers, especially when dealing with chronic situations, which require long-term coordination between the patient and the service provider. Receiving services from a specific healthcare provider for chronic health conditions is one of the main elements of the continuum of care [33]. Studies have shown that continuum of care is connected to greater glycemic control [34, 35], improvement of health-related quality of life [36], and lower odds for mortality in patients with T2DM [35].

Additionally, based on the results of the current study, patients in small cities reported a higher quality of services than those in rural areas. Aligning with our results, several studies have shown that patients with diabetes in rural areas are less likely to receive adequate and high-quality care compared to their non-rural counterparts [37, 38]. A systematic review has summarized several interventions targeting patients, professionals, and health systems to improve the quality of care for patients with diabetes in rural areas, including patient education, clinician education, and electronic patient registry [39]. Recent studies from LMICs also have reported the improvement of diabetes and/or hypertension care as a result of interventions such as patient education by health workers/nurses [40] and professionals’ and patients’ joint advocacy for health system reform to improve the access to medication and disease management/prevention services in rural areas [41].

### Implications for policy, practice, and research

The results of this study are crucial for enhancing health authorities’ understanding of the quality of healthcare services for patients with Type 2 Diabetes Mellitus (T2DM) and/or hypertension, along with identifying determinant factors. This knowledge is foundational for initiating improvements in service quality and addressing the specific needs of patients with chronic health conditions. A deep understanding of the healthcare service landscape for patients with chronic health conditions is deemed monumental. This understanding serves as the initial step towards implementing targeted interventions and strategies to enhance the overall quality of services provided to patients. It lays the groundwork for addressing challenges and optimizing care delivery. The emphasis of the World Health Organization (WHO) on universal health coverage and the management of chronic diseases, particularly in developing countries, aligns with the significance of this study’s results. The holistic views presented on the quality of services for individuals with T2DM and/or hypertension, encompassing both rural and urban areas in a Low- and Middle-Income Country (LMIC), contribute to global health priorities. In summary, the study’s implications extend to informing policy decisions, guiding practice improvements, and shaping the trajectory of future research endeavors. The holistic perspective provided by this research contributes to the ongoing global efforts to enhance healthcare services for individuals with chronic conditions, particularly in LMICs.

### Limitations

We acknowledge that there are some limitations to this study. First, the main health outcomes of T2DM and hypertension, such as Hemoglobin HA1c and blood pressure, were missed from patients’ medical records and, therefore, were not included in the data analysis. Second, the samples were patients with T2DM and/or hypertension who received healthcare services from the public sector and those who were visited by physicians in their private offices were not included in the study. As a result, we were not able to compare the SQ in the private and public sectors. Despite these limitations, this study could provide more insight into how SQ of T2DM and hypertension may be varied among patients with different characteristics and different geographical residencies.

## Conclusion

The results of the current study revealed that even though the primary health system has initiated delivering routine check-ups for patients with T2DM and/or hypertension in primary health centers a decade ago, there is a gap in the quality of services provided. While SQ scores across participant groups were generally average, significant weaknesses were identified in the availability of support groups, self-care training, and dietary counseling. Notably, higher SQ scores correlated with better self-care status, suggesting the importance of patient empowerment in improving care outcomes. Stability in healthcare providers was also highlighted as essential for continuity of care, particularly in managing chronic conditions like T2DM and hypertension. Notably, higher SQ scores correlated with better self-care status, suggesting the importance of patient empowerment in improving care outcomes. Furthermore, disparities in service quality between small cities and rural areas were evident, with rural populations facing greater challenges in accessing adequate care. Addressing these disparities requires targeted interventions such as patient and clinician education initiatives, as well as health system reforms to improve access to medication and disease management services in rural areas. Overall, enhancing service quality in primary healthcare settings necessitates a comprehensive approach that prioritizes patient empowerment, continuity of care, and equitable access to services, particularly for vulnerable populations in rural areas.

### Electronic supplementary material

Below is the link to the electronic supplementary material.

Supplementary Material 1

Supplementary Material 2

## Data Availability

The datasets used and/or analyzed during the current study are available from the corresponding author on reasonable request.

## Abbreviations

BMI: Body Mass Index
FBG: Mean Fasting Blood Glucose
SQ: Service Quality
SD: Standard Deviation
T2DM: Type2 Diabetes Mellitus
