# Correction: Service quality: perspective of people with type 2 diabetes mellitus and hypertension in rural and urban public primary healthcare centers in Iran

**DOI:** 10.1186/s12913-024-11172-z

**Published:** 2024-06-13

**Authors:** Shabnam Iezadi, Kamal Gholipour, Jabraeil Sherbafi, Sama Behpaie, Nazli Soltani, Mohsen Pasha, Javad Farahishahgoli

**Affiliations:** 1 Tabriz Health Services Management Research Center, Department of Health Policy and Management, School of Management and Medical Informatics, Tabriz University of Medical Sciences, Tabriz, Iran; 2 East Azerbaijan Provincial Health Centre, Tabriz University of Medical Sciences, Tabriz, Iran; 3 Student Research Committee, Department of Health Policy and Management, School of Management and Medical Informatics, Tabriz University of Medical Sciences, Tabriz, Iran

**Correction to: Iezadi et al. BMC Health Services Research (2024) 24:517**

10.1186/s12913-024-10854-y.

Due to a typesetting error in the Original Article, some parts of the Results section were placed in the Conclusion section.

The second paragraph of the Conclusion, which started with “*The findings regarding self-reported hypertension self-management status indicated that among individuals with hypertension without Type 2 Diabetes Mellitus (T2DM)…*” until the last sentence in the section, as well as Tables 3, 4, 5 and 6, should have been part of the Results.

The correct Results and Conclusion are as follows:

## Results

Among the 637 contacted patients, an impressive 561 individuals participated in the study, reflecting a robust response rate of 91.1%. The majority of participants were female (69%), hailing from metropolitan areas (36%), predominantly speaking Azeri (94%), unemployed (74%), lacking supplementary health insurance (65%), and reporting illiteracy (41%) (Table 1).

The anthropometric indices and biochemical characteristics of the participants revealed a predominant occurrence of overweight status. Notably, the mean Fasting Blood Glucose (FBG) levels in individuals with Type 2 Diabetes Mellitus (T2DM) were elevated, measuring 174.4 (73.57) in patients with T2DM without hypertension and 159.4 (65.46) in patients with both T2DM and Hypertension. Additional details regarding the participants’ anthropometric indices and biochemical characteristics can be found in Table 2.

The findings regarding self-reported hypertension self-management status indicated that among individuals with hypertension without Type 2 Diabetes Mellitus (T2DM), the majority adhered to the “regular use of prescription drugs” (approximately 94%). Conversely, “regular blood pressure measurement at home” was the least adhered-to item, with an adherence rate of around 61%. In contrast, among patients with both T2DM and hypertension, a substantial proportion reported adherence to a “recommended diet” (approximately 90%) and being “aware of the side effects of high blood pressure” (roughly 88%). The results of Fisher’s Exact Test and Independent Samples Test demonstrated no statistically significant relationship between hypertension self-management status and the presence of T2DM among individuals with hypertension, neither for sub-items nor for the total score. Comprehensive details on the hypertension self-management status of participants are presented in Table 3.

The self-reported Type 2 Diabetes Mellitus (T2DM) self-management status revealed that the majority of participants adhered to the “regular use of prescription drugs” (approximately 97%). Conversely, “regular glucose measurement at home” emerged as the least adhered-to items, with adherence rates of approximately 58% among patients with T2DM without hypertension and 47% among patients with both T2DM and hypertension. A comprehensive overview of the T2DM self-management status of patients is presented in Table 4.

Among all 13 dimensions of Service Quality (SQ), confidentiality and dignity exhibited the highest scores across all groups. The total SQ scores were 82.37 (12.19), 82.48 (12.45), and 81.69 (11.75) for hypertension, Type 2 Diabetes Mellitus (T2DM), and both conditions (Hypertension & T2DM), respectively. Notably, there were no statistically significant differences in total SQ scores between the groups (*P* = 0.780). Detailed results of SQ scores for each group are presented in Table 5.

The Multiple Regression model results unveiled several relationships with Service Quality (SQ) scores in different patient groups. Among individuals with hypertension and without diabetes, those with specific service providers demonstrated higher SQ scores (b = 7.03; *p* < 0.001) compared to those without specific service providers. Moreover, individuals in rural areas with hypertension and without diabetes exhibited lower SQ scores (b = -6.07; *p* < 0.05) than their urban counterparts.

In the group of patients with Type 2 Diabetes Mellitus (T2DM) and without hypertension, those residing in non-metropolitan cities reported higher SQ scores compared to patients in metropolitan areas (b = 5.09; *p* < 0.05). Additionally, a one-point increase in self-management total score was related with a 0.13-point decrease in SQ score (*P* < 0.05).

For people with both hypertension and T2DM, those with specific service providers demonstrated higher SQ scores (b = 8.32; *p* < 0.001) compared to those without specific service providers. Patients with both conditions who had a diabetes history of over 10 years exhibited higher SQ scores than those with less than two years of diabetes history (b = 4.47; *p* < 0.05).

## Conclusion

The results of the current study revealed that even though the primary health system has initiated delivering routine check-ups for patients with T2DM and/or hypertension in primary health centers a decade ago, there is a gap in the quality of services provided. While SQ scores across participant groups were generally average, significant weaknesses were identified in the availability of support groups, self-care training, and dietary counseling. Notably, higher SQ scores correlated with better self-care status, suggesting the importance of patient empowerment in improving care outcomes. Stability in healthcare providers was also highlighted as essential for continuity of care, particularly in managing chronic conditions like T2DM and hypertension. Notably, higher SQ scores correlated with better self-care status, suggesting the importance of patient empowerment in improving care outcomes. Furthermore, disparities in service quality between small cities and rural areas were evident, with rural populations facing greater challenges in accessing adequate care. Addressing these disparities requires targeted interventions such as patient and clinician education initiatives, as well as health system reforms to improve access to medication and disease management services in rural areas. Overall, enhancing service quality in primary healthcare settings necessitates a comprehensive approach that prioritizes patient empowerment, continuity of care, and equitable access to services, particularly for vulnerable populations in rural areas.

The Original Article has been corrected.

