# Supplementary material for: Service quality: perspective of people with type 2 diabetes mellitus and hypertension in rural and urban public primary healthcare centers in Iran
Source: BMC Health Serv Res. 2024 Apr 24;24:517. doi: 10.1186/s12913-024-10854-y (PMC11044473; doi:10.1186/s12913-024-10854-y)
Supplement: Supplementary file 1 — Supplementary Material 1 [file 12913_2024_10854_MOESM1_ESM.docx]

**Supporting information:**

**S table1: study participants self-reported care provider types and care conditions**

|  | | HBP | | T2DM | | HBP & T2DM | |
| --- | --- | --- | --- | --- | --- | --- | --- |
| Characteristics | | N | % | N | % | N | % |
| **Seeing Health expert** | yes | 114 | 64.8 | 101 | 61.6 | 153 | 69.9 |
|  | no | 62 | 35.2 | 63 | 38.4 | 66 | 30.1 |
| **Seeing community health worker** | yes | 4 | 2.3 | 6 | 3.7 | 7 | 3.2 |
|  | no | 172 | 97.7 | 158 | 96.3 | 212 | 96.8 |
| **Seeing general practitioner** | yes | 54 | 30.7 | 52 | 31.7 | 69 | 31.5 |
|  | no | 122 | 69.3 | 112 | 68.3 | 150 | 68.5 |
| **Seeing cardiologist** | yes | 5 | 2.8 | 9 | 5.5 | 10 | 4.6 |
|  | no | 171 | 97.2 | 155 | 94.5 | 209 | 95.4 |
| **Seeing medical specialist** | yes | 147 | 83.5 | 133 | 81.1 | 186 | 84.9 |
|  | no | 29 | 16.5 | 31 | 18.9 | 33 | 15.1 |
| **Seeing nurses** | yes | 8 | 4.5 | 10 | 6.1 | 13 | 5.9 |
|  | no | 167 | 94.9 | 154 | 93.9 | 206 | 94.1 |
| **Seeing Traditional healer** | yes | 53 | 30.1 | 19 | 11.6 | 61 | 27.9 |
|  | no | 123 | 69.9 | 145 | 88.4 | 158 | 72.1 |
| **Using Herbal Shopping services** | yes | 32 | 18.2 | 45 | 27.4 | 70 | 32.0 |
|  | no | 144 | 81.8 | 119 | 72.6 | 149 | 68.0 |
| **Seeing Internist** | yes | 30 | 17.0 | 33 | 20.1 | 42 | 19.2 |
|  | no | 146 | 83.0 | 131 | 79.9 | 177 | 80.8 |
| **Endocrinologist** | yes | 10 | 5.7 | 38 | 23.2 | 39 | 17.8 |
|  | no | 166 | 94.3 | 126 | 76.8 | 180 | 82.2 |
| **Receive care from the private sector** | yes | 62 | 35.6 | 61 | 39.9 | 79 | 36.1 |
|  | no | 112 | 64.4 | 92 | 60.1 | 140 | 63.9 |
| **Having continuity of care1** | yes | 99 | 58.9 | 91 | 60.3 | 98 | 45.6 |
|  | no | 69 | 41.1 | 60 | 39.7 | 117 | 54.4 |

1 Seeing the same care provider for care during last year

**S table 2:self-reported care condition, complications and limitation in study participants**

|  | | HBP | | T2DM | | HBP & T2DM | |
| --- | --- | --- | --- | --- | --- | --- | --- |
| Characteristics | | N | % | N | % | N | % |
| Duration of T2DM awareness | Under 2 years |  |  | 50 | 24.3 | 31 | 18.1 |
|  | 2 – 5 years |  |  | 46 | 22.3 | 41 | 24.0 |
|  | 5 – 10 years |  |  | 59 | 28.6 | 46 | 26.9 |
|  | Over 10 years |  |  | 51 | 24.8 | 53 | 31.0 |
| The use of medication to control T2DM | yes |  |  | 153 | 96.8 | 213 | 97.3 |
|  | no |  |  | 5 | 3.2 | 6 | 2.7 |
| use diet to control T2DM | no |  |  | 41 | 25.9 | 44 | 20.1 |
|  | Yes |  |  | 117 | 74.1 | 175 | 79.9 |
| The use of Exercise to control T2DM | yes |  |  | 106 | 64.2 | 149 | 67.7 |
|  | no |  |  | 52 | 31.5 | 70 | 31.8 |
| DM2_DM_Mdside | no |  |  | 28 | 35.4 | 39 | 34.5 |
|  | yes |  |  | 51 | 64.6 | 74 | 65.5 |
| B5_DM_side | no |  |  | 74 | 61.7 | 87 | 50.6 |
|  | yes |  |  | 46 | 38.3 | 85 | 49.4 |
| Limitations in walking | yes |  |  | 17 | 14.3 | 36 | 21.2 |
|  | no |  |  | 102 | 85.7 | 134 | 78.8 |
| Limitations in food consumption | yes |  |  | 46 | 38.7 | 79 | 46.5 |
|  | no |  |  | 73 | 61.3 | 91 | 53.5 |
| Limitations in Talking | no |  |  | 118 | 100.0 | 166 | 2.4 |
|  | yes |  |  |  |  | 4 | 97.6 |
| BP2_BP_MD | no | 18 | 10.3 |  |  | 35 | 15.9 |
|  | yes | 157 | 89.7 |  |  | 185 | 84.1 |
| Duration of BP awareness | Under 2 years | 33 | 21.3 |  |  | 32 | 21.6 |
|  | 2 – 5 years | 51 | 32.9 |  |  | 43 | 29.1 |
|  | 5 – 10 years | 39 | 25.2 |  |  | 48 | 32.4 |
|  | Over 10 years | 32 | 20.6 |  |  | 25 | 16.9 |
| The use of medication to control BP | yes | 169 | 96.0 |  |  | 209 | 95.4 |
|  | no | 7 | 4.0 |  |  | 10 | 4.6 |
| use diet to control BP | yes | 83 | 47.2 |  |  | 142 | 64.8 |
|  | no | 93 | 52.8 |  |  | 77 | 35.2 |
| The use of Exercise to control BP | yes | 40 | 22.7 |  |  | 52 | 23.7 |
|  | no | 136 | 77.3 |  |  | 167 | 76.3 |
| Doing exercise | No | 89 | 52.7 |  |  | 109 | 51.7 |
|  | Sometime | 49 | 29.0 |  |  | 61 | 28.9 |
|  | Almost | 17 | 10.1 |  |  | 26 | 12.3 |
|  | Every time | 14 | 8.3 |  |  | 15 | 7.1 |
| Number of meals per day | 2.00 | 10 | 6.0 |  |  | 10 | 4.7 |
|  | 3.00 | 144 | 86.2 |  |  | 177 | 82.3 |
|  | 4.00 | 9 | 5.4 |  |  | 17 | 7.9 |
|  | 5.00 | 3 | 1.8 |  |  | 10 | 4.7 |
|  | 6.00 | 1 | .6 |  |  | 1 | .5 |
| Use of fast foods | .00 | 122 | 89.7 |  |  | 171 | 95.5 |
|  | 1.00 | 9 | 6.6 |  |  | 8 | 4.5 |
|  | 2.00 | 2 | 1.5 |  |  |  |  |
|  | 3.00 | 3 | 2.2 |  |  |  |  |
| Smoking | no | 158 | 89.8 | 87 | 89.7 | 175 | 79.9 |
|  | yes | 18 | 10.2 | 10 | 10.3 | 44 | 20.1 |
| Hookah consumption | no | 161 | 92.0 | 86 | 96.6 | 186 | 84.9 |
|  | yes | 14 | 8.0 | 3 | 3.4 | 33 | 15.1 |
| Existence of complications of BP | no | 138 | 89.0 |  |  | 177 | 84.3 |
|  | yes | 17 | 11.0 |  |  | 33 | 15.7 |

**S Table 3: Results of univariate linear Regression Analysis for Variables Related to total *SQ score***

|  | | **BP** | | | | **DM** | | | | **BP & DM** | | | |
| --- | --- | --- | --- | --- | --- | --- | --- | --- | --- | --- | --- | --- | --- |
|  |  | **B** | **95% CI** | | **P** | **B** | **95% CI** | | **P** | **B** | **95% CI** | | **P** |
|  |  |  | **LB** | **UB** |  |  | **LB** | **UB** |  |  | **LB** | **UB** |  |
| BMI |  | 0.06 | -0.331 | 0.45 | 0.764 | -0.54 | -0.96 | -0.1 | 0.013 | -0.05 | -0.43 | 0.326 | 0.796 |
| Weight |  | 0.14 | -0.013 | 0.29 | 0.072 | -0.19 | -0.33 | -0 | 0.01 | 0.03 | -0.1 | 0.163 | 0.663 |
| FBS |  | 0.04 | -0.073 | 0.15 | 0.503 | -0.01 | -0.04 | 0.03 | 0.778 | -0.01 | -0.04 | 0.023 | 0.568 |
| HbA1c |  | -20 | -63.68 | 24.6 | 0.197 | 0.03 | -0.14 | 0.2 | 0.717 | -0.18 | -0.55 | 0.197 | 0.348 |
| Systolic BP |  | 1.94 | -0.167 | 4.04 | 0.071 | 0.95 | -1.71 | 3.6 | 0.48 | -1.78 | -3.59 | 0.022 | 0.053 |
| Diastolic BP |  | -1.34 | -4.439 | 1.76 | 0.394 | 0.09 | -0.35 | 0.53 | 0.684 | -4.36 | -6.83 | -1.895 | 0.001 |
| Age |  | 0.03 | -0.125 | 0.19 | 0.676 | 0.1 | -0.05 | 0.26 | 0.191 | -0.05 | -0.21 | 0.1 | 0.499 |
| Sex | Male | 1 |  |  |  | 1 |  |  |  | 1 |  |  |  |
|  | Female | -0.4 | -4.51 | 3.68 | 0.841 | 0.18 | -3.81 | 4.17 | 0.929 | -1.37 | -4.84 | 2.107 | 0.439 |
| Residence | Metropolitan | 1 |  |  |  | 1 |  |  |  | 1 |  |  |  |
|  | City | 2.99 | -1.50 | 7.48 | 0.190 | 6.47 | 2.00 | 10.93 | 0.005 | 4.82 | 1.13 | 8.52 | 0.011 |
|  | Village | -1.44 | -5.93 | 3.05 | 0.528 | -0.16 | -4.7 | 4.42 | 0.944 | -0.23 | -3.99 | 3.52 | 0.902 |
| Employment status | No* |  |  |  |  | 1 |  |  |  | 1 |  |  |  |
|  | Yes | 0.79 | -3.269 | 4.84 | 0.702 | -0.22 | -4.45 | 4.01 | 0.917 | 0.825 | -2.4 | 4.052 | 0.615 |
| Having health insurance | No* |  |  |  |  |  |  |  |  |  |  |  |  |
|  | Yes | 0.78 | -7.95 | 9.51 | 0.86 | 9.6 | -2.8 | 22 | 0.128 | -3.92 | -15.6 | 7.783 | 0.51 |
| Education level | Illiterate* | 1 |  |  |  | 1 |  |  |  | 1 |  |  |  |
|  | Under diploma | -1 | -5.149 | 3.08 | 0.621 | 0.15 | -4.04 | 4.33 | 0.945 | 1.255 | -2.07 | 4.578 | 0.458 |
|  | Diploma | 0.88 | -5.227 | 6.98 | 0.777 | 3.52 | -4.57 | 11.6 | 0.391 | 1.051 | -5.76 | 7.865 | 0.762 |
|  | Tertiary | -1.7 | -8.491 | 5.07 | 0.62 | -1.45 | -9.53 | 6.64 | 0.724 | 6.763 | -1.29 | 14.82 | 0.099 |
| Receive care from the private sector | No* | 1 |  |  |  | 1 |  |  |  | 1 |  |  |  |
|  | Yes | -2.2 | -5.959 | 1.63 | 0.262 | -1.42 | -5.58 | 2.74 | 0.501 | -4.34 | -7.55 | -1.14 | 0.008 |
| Having continuity of care1 | No* | 1 |  |  |  | 1 |  |  |  | 1 |  |  |  |
|  | Yes | 5.88 | 2.231 | 9.53 | 0.002 | 4.68 | 0.515 | 8.84 | 0.028 | 7.748 | 4.75 | 10.75 | <0.001 |
| **Smoke/hookah consumption** | No* | 1 |  |  |  | 1 |  |  |  | 1 |  |  |  |
|  | Yes | 11.1 | 1.195 | 21 | 0.028 | -4.82 | -11.1 | 1.45 | 0.131 | 0.978 | -5.91 | 7.87 | 0.78 |
| Existence of complications of T2DM | No* |  |  |  |  | 1 |  |  |  | 1 |  |  |  |
|  | Yes |  |  |  |  | -0.62 | -4.99 | 3.76 | 0.78 | -1.75 | -4.89 | 1.397 | 0.274 |
| Existence of complications of BP | No* | 1 |  |  |  |  |  |  |  | 1 |  |  |  |
|  | Yes | 0.95 | -5.149 | 7.05 | 0.759 |  |  |  |  | 2.666 | -1.58 | 6.911 | 0.217 |
| Duration of BP awareness | Under 2 years | 1 |  |  |  |  |  |  |  | 1 |  |  |  |
|  | 2 – 5 years | 2.35 | -2.354 | 7.05 | 0.326 |  |  |  |  | -4.85 | -9.25 | -0.44 | 0.031 |
|  | 5 – 10 years | 0.08 | -4.989 | 5.14 | 0.977 |  |  |  |  | -0.04 | -4.29 | 4.202 | 0.984 |
|  | Over 10 years | 0.33 | -5.049 | 5.71 | 0.904 |  |  |  |  | 1.355 | -2.71 | 5.418 | 0.512 |
| Duration of T2DM awareness | Under 2 years |  |  |  |  | 1 |  |  |  | 1 |  |  |  |
|  | 2 – 5 years |  |  |  |  | 1.29 | -3.81 | 6.39 | 0.619 | -3.86 | -8.28 | 0.558 | 0.086 |
|  | 5 – 10 years |  |  |  |  | -4.35 | -9.3 | 0.61 | 0.085 | 0.492 | -3.64 | 4.62 | 0.814 |
|  | Over 10 years |  |  |  |  | -1.99 | -7.99 | 4.01 | 0.513 | 3.031 | -1.26 | 7.324 | 0.165 |

* Reference category

**S Table 4: Results of univariate linear Regression Analysis for Self-management condition to total *SQ score***

|  | | **BP** | | | | **DM** | | | | **BP & DM** | | | |
| --- | --- | --- | --- | --- | --- | --- | --- | --- | --- | --- | --- | --- | --- |
|  |  | **B** | **95% CI** | | **P** | **B** | **95% CI** | | **P** | **B** | **95% CI** | | **P** |
|  |  |  | **LB** | **UB** |  |  | **LB** | **UB** |  |  | **LB** | **UB** |  |
| T2DM Self-management Total score |  |  |  |  |  | 11.15 | 2.198 | 20.1 | 0.015 | 3.89 | -3.93 | 11.71 | 0.328 |
| BP Self-management Total score |  | 8.39 | -0.518 | 17.3 | 0.065 |  |  |  |  | 4.443 | -3.58 | 12.47 | 0.276 |
| Family history of high blood pressure | No* | 1 |  |  |  |  |  |  |  | 1 |  |  |  |
|  | Yes | 0.92 | -2.954 | 4.79 | 0.641 |  |  |  |  | -2.61 | -5.93 | 0.714 | 0.123 |
| See the caregiver on time and regularly | No* | 1 |  |  |  |  |  |  |  | 1 |  |  |  |
|  | Yes | 4.63 | 0.456 | 8.8 | 0.03 |  |  |  |  | 1.763 | -2.08 | 5.604 | 0.367 |
| Regular blood pressure measurement at home | No* | 1 |  |  |  |  |  |  |  | 1 |  |  |  |
|  | Yes | 2.71 | -1.013 | 6.42 | 0.153 |  |  |  |  | 3.38 | 0.26 | 6.505 | 0.034 |
| See your doctor regularly despite your blood pressure being normal | No* | 1 |  |  |  |  |  |  |  | 1 |  |  |  |
|  | Yes | 4.21 | 0.064 | 8.37 | 0.047 |  |  |  |  | -0.23 | -3.79 | 3.323 | 0.898 |
| Regular use of prescription drugs | No* | 1 |  |  |  |  |  |  |  | 1 |  |  |  |
|  | Yes | 1.69 | -5.809 | 9.18 | 0.658 |  |  |  |  | -1.57 | -9.96 | 6.815 | 0.712 |
| Adhere to the recommended diet | No* | 1 |  |  |  |  |  |  |  | 1 |  |  |  |
|  | Yes | -1.2 | -6.595 | 4.18 | 0.658 |  |  |  |  | 0.572 | -4.26 | 5.404 | 0.816 |
| Knowing the side effects of high blood pressure | No* | 1 |  |  |  |  |  |  |  | 1 |  |  |  |
|  | Yes | -1.7 | -7.05 | 3.72 | 0.542 |  |  |  |  | -0.12 | -5.35 | 5.11 | 0.965 |
| Family history of T2DM | No* |  |  |  |  | 1 |  |  |  | 1 |  |  |  |
|  | Yes |  |  |  |  | 1.26 | -2.58 | 5.1 | 0.518 | -0.62 | -3.79 | 2.545 | 0.699 |
| See the caregiver on time and regularly | No* |  |  |  |  | 1 |  |  |  | 1 |  |  |  |
|  | Yes |  |  |  |  | 3.02 | -1.59 | 7.64 | 0.198 | 2.053 | -1.91 | 6.016 | 0.308 |
| Regular Blood glucose measurement at home | No* |  |  |  |  | 1 |  |  |  | 1 |  |  |  |
|  | Yes |  |  |  |  | 2.7 | -1.18 | 6.57 | 0.171 | 2.413 | -0.7 | 5.53 | 0.128 |
| See your doctor regularly despite your blood glucose being normal | No* |  |  |  |  | 1 |  |  |  | 1 |  |  |  |
|  | Yes |  |  |  |  | 4.97 | 0.979 | 8.97 | 0.015 | 1.026 | -2.44 | 4.494 | 0.561 |
| Regular use of prescription drugs | No* |  |  |  |  | 1 |  |  |  | 1 |  |  |  |
|  | Yes |  |  |  |  | -7.22 | -19.7 | 5.25 | 0.254 | -1.04 | -9.39 | 7.314 | 0.807 |
| Adhere to the recommended diet | No* |  |  |  |  | 1 |  |  |  | 1 |  |  |  |
|  | Yes |  |  |  |  | 6.39 | 1.142 | 11.6 | 0.017 | -1.21 | -5.7 | 3.27 | 0.594 |
| Knowing the side effects of T2DM | No* |  |  |  |  | 1 |  |  |  | 1 |  |  |  |
|  | Yes |  |  |  |  | 0.38 | -5.49 | 6.25 | 0.899 | -0.07 | -4.99 | 4.847 | 0.977 |

* Reference category
